# Advancing sustainable agriculture: the potential of seaweed-derived bio pesticides from marine biomass

**DOI:** 10.1186/s40643-025-00849-w

**Published:** 2025-03-22

**Authors:** Sasikala Sekar, Sivakamavalli Jeyachandran, Jayant Giri, Mohammed Aman

**Affiliations:** 1 Department of Food and Nutrition, Queens Mary College, Chennai, Tamil Nadu India; 2 Lab in Biotechnology and Biosignal Transduction, Department of Orthodontics, Saveetha Dental College and Hospital, Saveetha Institute of Medical and Technical Sciences (SIMATS), Saveetha University-77, Chennai, Tamil Nadu India; 3 Division of Research and Development, Lovely Professional University, Phagwara, India; 4 Centre for Research Impact & Outcome, Chitkara University Institute of Engineering and Technology, Chitkara University, Rajpura, 140401 Punjab India; 5 Department of Industrial Engineering, College of Engineering, University of Business and Technology, 21448 Jeddah, Saudi Arabia; 6 Department of Mechanical Engineering, Yeshwantrao Chavan College of Engineering, Nagpur, India

**Keywords:** Seaweeds, Biopesticides, Marine biomass, Bioactive compounds, Sustainable agriculture

## Abstract

The exploitation of marine biomass, particularly seaweeds, presents significant opportunities for sustainable biopesticide production. Seaweeds, or macroalgae are diverse photosynthetic organisms found in marine environments, rich in bioactive compounds such as terpenoids, polyphenols, and sulphated polysaccharides. These compounds exhibit potent pesticide properties, offering environmentally friendly alternatives to conventional chemical pesticides. The biopesticides derived from seaweeds are biodegradable, exhibit low toxicity to non-target organisms, and align with the principles of environmental sustainability. The growing demand for organic and sustainable agricultural products further underscores the economic potential of seaweed-based biopesticides. However, challenges such as scalability of production, extraction efficiency, and standardization of bioactivity must be addressed to realize this potential fully. Advances in biorefinery approaches, extraction technologies, and formulation techniques are critical to overcoming these challenges. Additionally, ongoing research into the synergistic effects of different seaweed compounds and the development of innovative delivery systems will enhance the efficacy and commercial viability of seaweed-derived biopesticides. This review highlights the promising role of seaweeds in biopesticide production, emphasizing the need for continued research and interdisciplinary collaboration to integrate these natural compounds into sustainable agricultural practices.

**Graphical Abstract:**

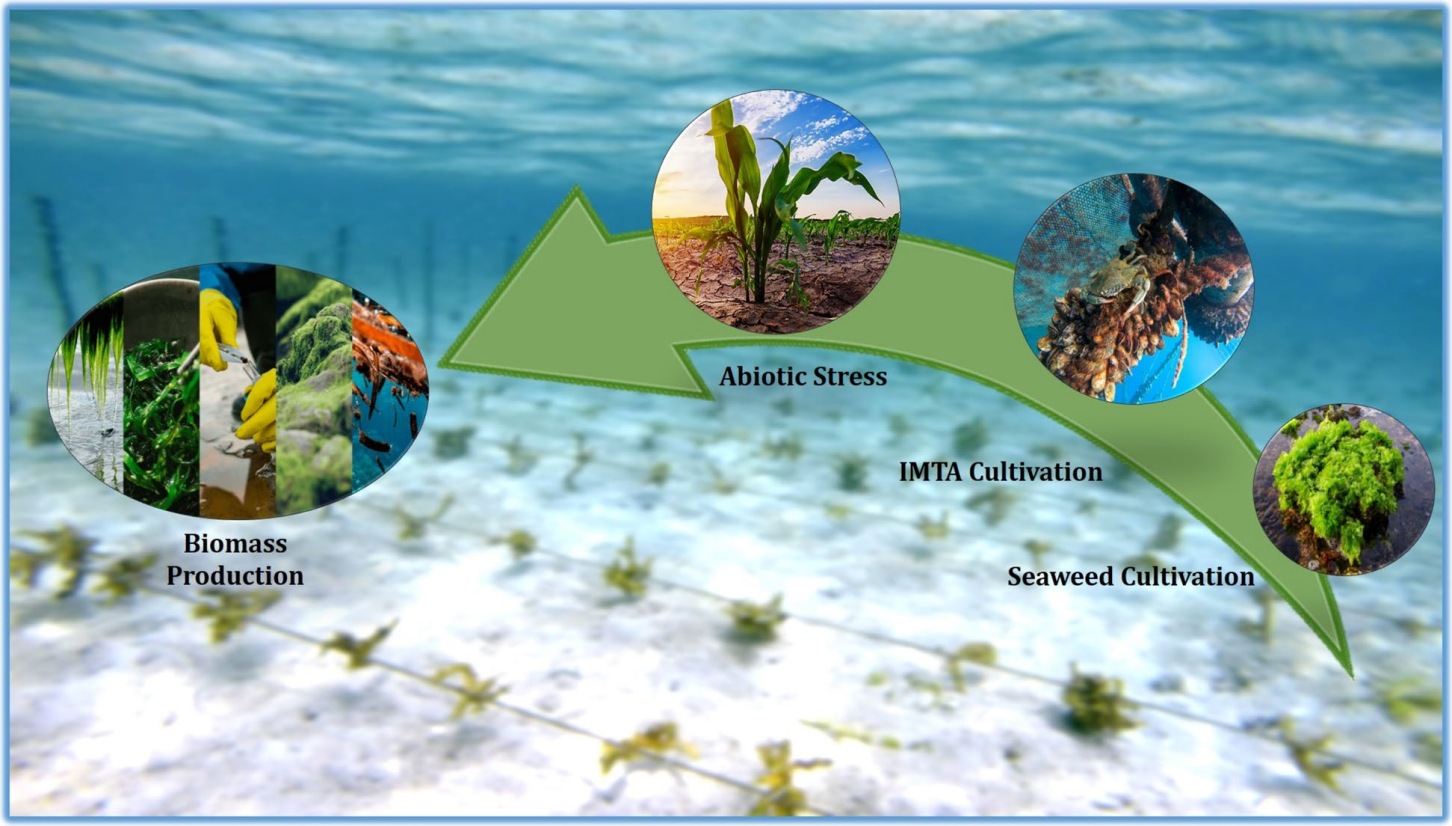

## Introduction

Marine environments encompass more than 70% of Earth's surface, hosting approximately half of the world's biodiversity among its myriad species. Algae live in the upper oceanic layer, which makes up two thirds of the earth (Gomez-Zavaglia et al. 2019). The term "seaweed," sometimes known as "macroalgae" refers to a broad group of organisms with various traits and applications. Natural substances obtained from marine species have been essential for everyday nourishment, act as sustainable source of alternative nutrition (Biris-Dorhoi et al. 2020) and prescribed for prevention of illness throughout history (Subbiah et al. 2022). Currently, the seaweed industry is characterized by a high species concentration, notable regional variations, and rapid expansion. The amount of seaweed that has been grown were increased by an incredible thousandfold since 1950 (Cai 2021).

In 2021, global seaweed farming reached 35.2 million tons (live weight) (UNCTAD and FAO 2023) the seaweed industry’s market value is forecasted to increase seven-fold, to $85 billion by 2026. Out of 12,000 species, world seaweed production is concentrated in only five broad groups, and just 27 seaweed species items were reported cultivated in 2019 (Cai et al. 2021). Geographically, the seaweed industry is dominated by Asia (mostly East and South Asia) with 99.5 per cent (35 million tons) of all production by volume in 2021 (FAO 2023). In today's world, as marine resources continue to decline, there is an opportunity to utilize resources such as seaweed, which can be easily replenished and may even aid in the regeneration of the ecosystems that support them. (World Bank 2023a). Enhanced sustainable seaweed production and improved value chains can provide significant benefits for both the environment and the economy.

Seaweeds are being used as the raw materials for many industrial productions like agar, alginate and carrageenan but they continue to be widely consumed as food in Asian countries (Sultana 2019). Trade in seaweed and innovative blue food commodities is expected to become more significant as a result of climate change's danger to the sustainability of many local crops and the regularity of harvests (UNCTAD and FAO 2023). The seaweed business has grown significantly thus far, providing a wide range of prospects for future expansion, particularly for women who are increasingly involved in labor—intensive production and processing. Since more seaweed production may help to the people, environment, and an economy, these economic prospects should be taken advantage to the fullest extent possible. Attention to marine resources has been accelerated the exploration and utilization of green seaweeds for greater economic value (Xu et al. 2023).

Over the past decade, the marine ecosystem has garnered considerable attention from researchers and industrialists due to the presence of organisms producing compounds with notable biological activity. Macroalgae, a diverse group of photosynthetic organisms inhabiting marine waters, particularly stand out as promising subjects for research (Pereira 2021). Due to the increase in industrial demand for new bio sourced molecules, several types of biomasses are being exploited for the identification of bioactive metabolites and techno-functional biomolecules that are suitable for the subsequent uses in cosmetic, food and pharmaceutical fields (Hentali 2020).

Seaweed plays the crucial role for marine ecosystems because it provides an environment and food source for a wide range of aquatic creatures (Satpadi et al. 2022). It also provides tremendous deal of promise for commercialization when used as an organic fertilizer in agriculture to make up for plant nutrients such as nitrogen, phosphorus, and potassium deficiencies (Raghunandan et al. 2019). The main role of biopesticide products is marketed either to fight leaf diseases, root diseases, or fruit storage diseases (Lahlali et al., 2024). Great natural alternative could be seaweeds, which have nourishment property, yet performs pesticide role and will be still biodegradable. This review paper mainly focuses on opportunities and challenges in biopesticide production using marine biomass seaweeds.

## Definition of marine biomass (seaweeds)

Marine seaweed is a type of algae that can perform photosynthesis, which produces oxygen and absorbs carbon dioxide, with the support of a class of green pigments known as chlorophyll. Algae are categorized as macroalgae, which are smaller than microalgae and only visible under a microscope. Macroalgae can reach a maximum depth of 65 m and are more diverse in the oceans. For this reason, "algae" is often used to refer to "marine macroalgae or seaweeds." (Pereira 2021). Seaweed is one form of marine material that has demonstrated inherent biofuel potential (Rony et al. 2024).

### Biorefinery approaches to seaweed biomass

**Fig. 1:**
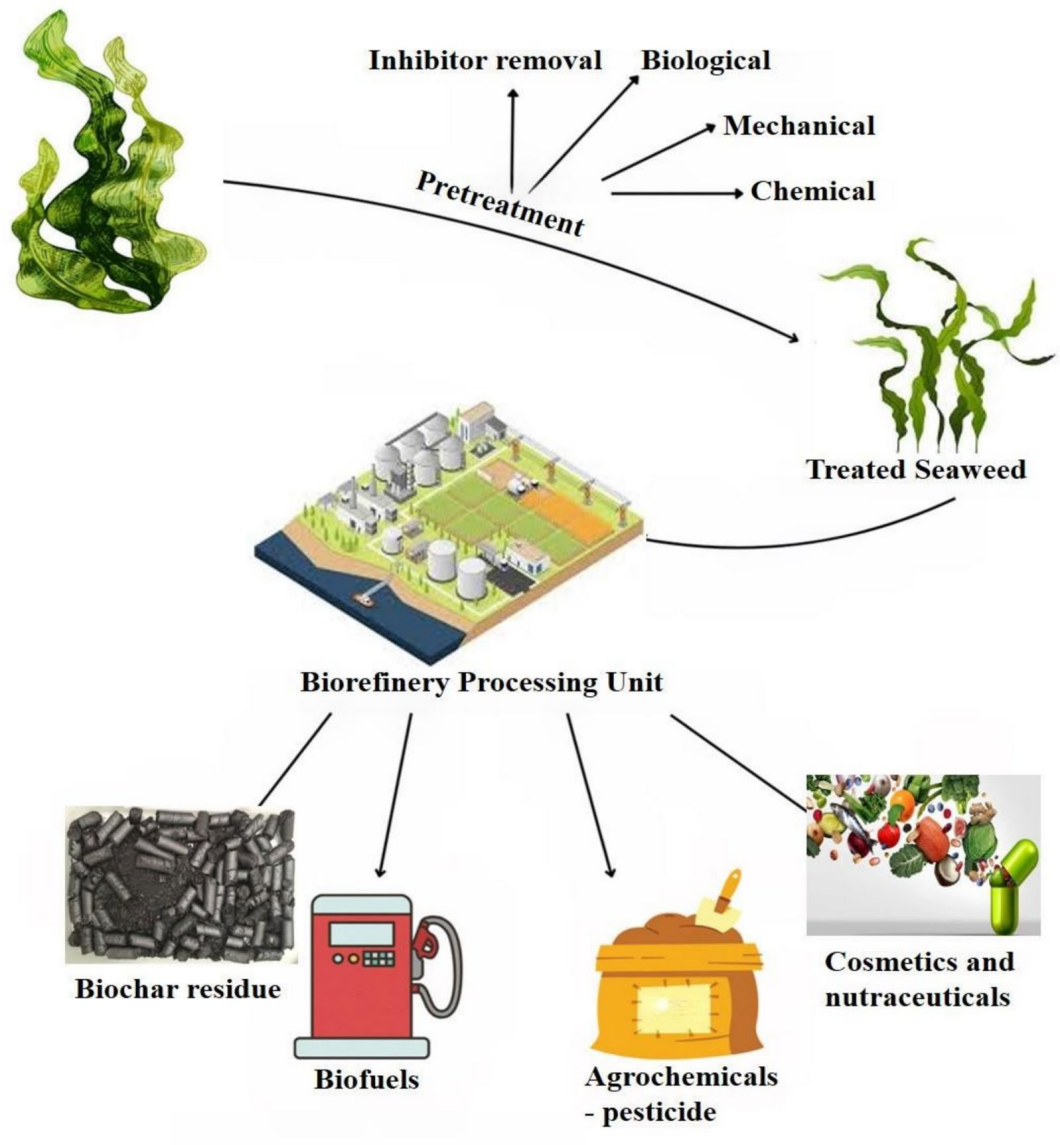
Biorefinery process of pesticide production from marine seaweeds

Biorefinery approaches to utilizing seaweed biomass focus on integrating the extraction of bioactive compounds for potential use as biopesticides along with other bioproducts and biofuels. The entire potential of seaweed can be realized by using a biorefinery concept, which will minimize waste and maximize product yield. In seaweed biorefineries, different procedures like fermentation, extraction, and purification would be used to separate chemicals that have pesticidal qualities (Fig. 1). The benevolent and biodegradable characteristics of these chemicals set them apart from synthetic insecticides. The adaptability of seaweed is highlighted by research in arena of biorefinery, which suggests its usage in food and fuel industries as part of an integrated resource management strategy (Siller-Sánchez et al., 2018).

## Bioactive compounds and pest management

Seaweeds, with their diverse array of species, offers a rich source of bioactive compounds that have potential uses in pest management in agriculture. As a source of biopesticides, different types of seaweeds—specifically brown, red, and green varieties—have shown pest-repellent properties that can be harnessed for agricultural and horticultural applications (Costa et al. 2019; Lomartire and Goncalves 2022). Researchers have identified bioactive compounds in seaweeds, such as *Ulva ohnoi* and *Derbesia tenuissima*, that can potentially be utilized as effective biopesticides. The controlled land-based techniques used to grow these seaweeds enable them as like biomass to be produced in a consistent amount and quality. The commercial manufacture of biopesticides depends on this uniformity since it guarantees the extracted compounds' efficacious and dependable pesticidal qualities. Because these are biodegradable and environmentally benign, these bioactive chemicals could act as a sustainable substitute for chemical pesticides (Mata et al. 2016).

### Challenges and potential

While the scenarios of using seaweed biomass for pesticide production are promising, several challenges need addressing to make this a viable alternative. These challenges include scalability of production, extraction efficiencies, and the economic viability of the process. There is also the issue of standardizing the bioactivity of the extracts, as natural products can vary significantly in their composition and efficacy. Addressing these issues requires innovative technological solutions and further research into cultivation methods, extraction techniques, and bioactivity assays. Nevertheless, the potential environmental benefits of reducing synthetic pesticide use present a compelling case for continued investment and research in this field (Popp et al. 2013). Even though algal aquaculture technology has advanced significantly over the past 70 years, particularly in Asia, America, and Europe, there is still much to be done to advance both the science and the associated societal acceptance. Creating strains that are resistant to heat, grow quickly, produce a lot of compounds of interest, are resistant to morbidity, and have the ability to prevent fouling is one of the biggest challenges. Another is creating effective and affordable hatcheries that can withstand storms in the open ocean (Moon et al. 2019). Research is still being done to better understand how chemicals generated from seaweed interact with human physiology and the environment, develop novel formulations, and maximize their usage in biomedical and pharmaceutical applications. Seaweed exhibits good biocompatibility in a range of contexts, including nutritional advantages, biomedical applications and environment sustainable products. Realizing its full potential while maintaining environmental sustainability and safety for human use requires ongoing study and ethical harvesting methods (Cotas et al. 2024). Seaweed-based biopesticides are gaining popularity and are the subject of increasing study; however, there are still issues with extraction method standardization, formulation stability, manufacturing scalability, and regulatory approval procedures. It will be essential to overcome these obstacles in order to promote and commercialize adoption (Hentali et al. 2020).

### Alternative bioproducts from seaweed

**Fig. 2:**
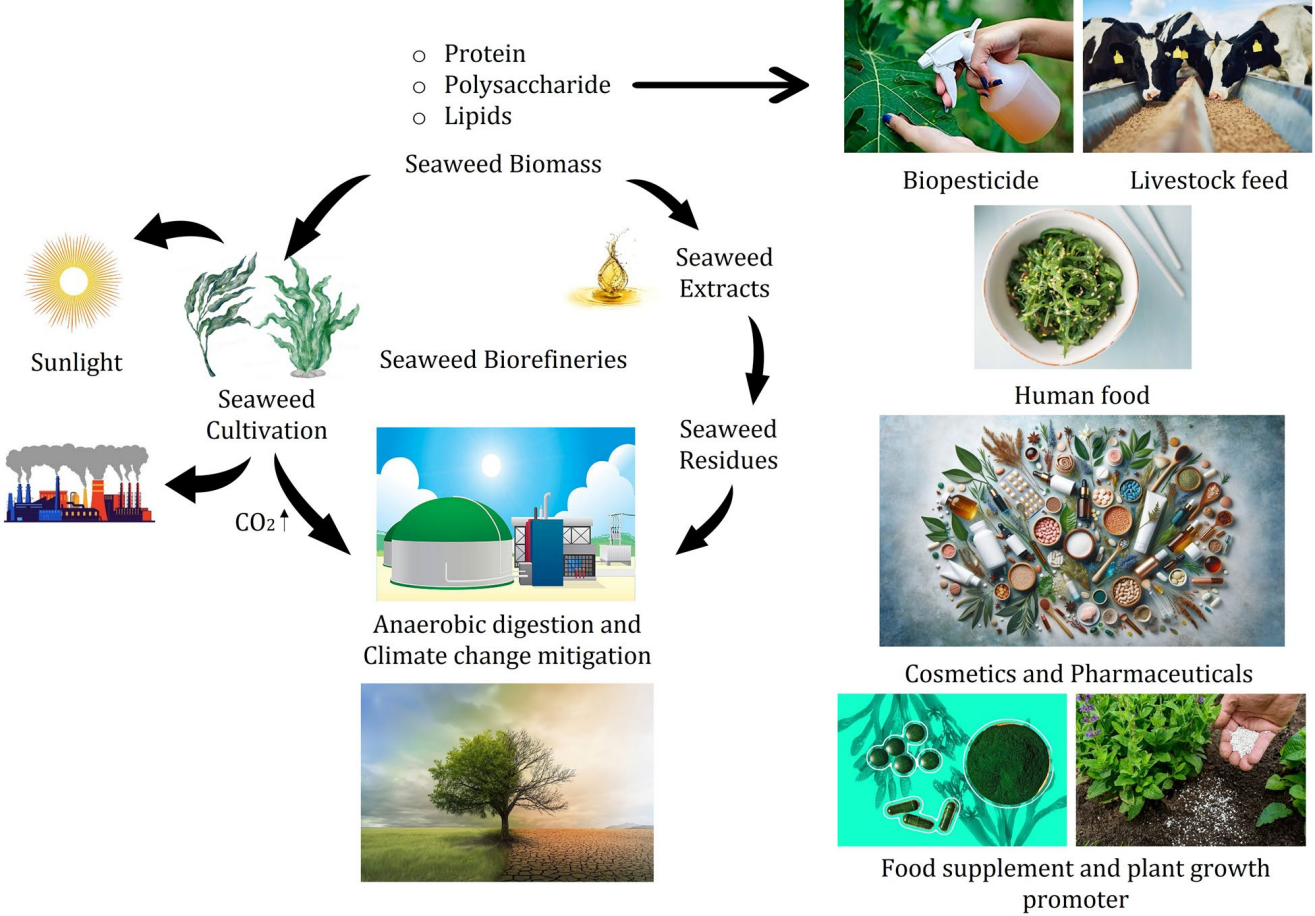
Schematic diagram of biopesticides processing mechanism from various seaweeds and its benefits in versatile

The utilization of seaweed biomass extends beyond pesticide production to include other valuable bioproducts like biodiesel and alginate. This diversification can provide economic benefits and increase the sustainability of seaweed cultivation practices. For instance, the valorisation of Caribbean Sargassum biomass not only addresses the environmental issues caused by its overabundance but also explores its potential in producing high-value products. Such initiatives can create new revenue streams for communities affected by Sargassum influxes while contributing to the development of sustainable bioproduct industries (Fig. 2). These efforts underline the broader applicability and economic potential of seaweed biomass in the bioeconomy (Sierra et al. 2022). Utilizing seaweeds to produce pesticides and repellents against mosquito species and as a source of environmentally friendly materials is an additional strategy (Bibi et al. 2020). Numerous studies have demonstrated the health advantages of seaweed polysaccharides, such as carrageenans and alginates, as well as their assistance for the production of biopolymeric films and biodegradable packaging. Ecosystems and aquatic life are impacted by the pervasiveness of plastics and microplastics in the ocean, which presents serious environmental challenges. Consequently, it is critical to use biopolymers made from seaweed in a sustainable manner. By using biodegradable substitutes for plasticizers, this method can assist safeguard the environment (Lomartine et al., 2022). The organic, nutraceutical and biofertilizer industries have shown interest in seaweeds as a source of macronutrients, micronutrients, and bioactive components (Cherry et al. 2019). The biomedical application of sea weed depend on its biocompatibility, compounds derived from seaweed, like carrageenan and alginate. Because of their biodegradability and gel- forming properties, they are utilized in tissue engineering, medication delivery systems, and wound dressings (Pacheco-Quito et al. 2020). Applications of seaweeds in biotechnology are numerous. Species in the phylum Rhodophyta, class Phaeophyceae, phylum Ochrophyta, and phylum Chlorophyta are valuable to the food, cosmetic, pharmaceutical, and nutraceutical sectors because of the range of bioactive substances in their composition (Lomartire et al. 2022).

### Importance of seaweeds in biopesticides production

**Fig. 3:**
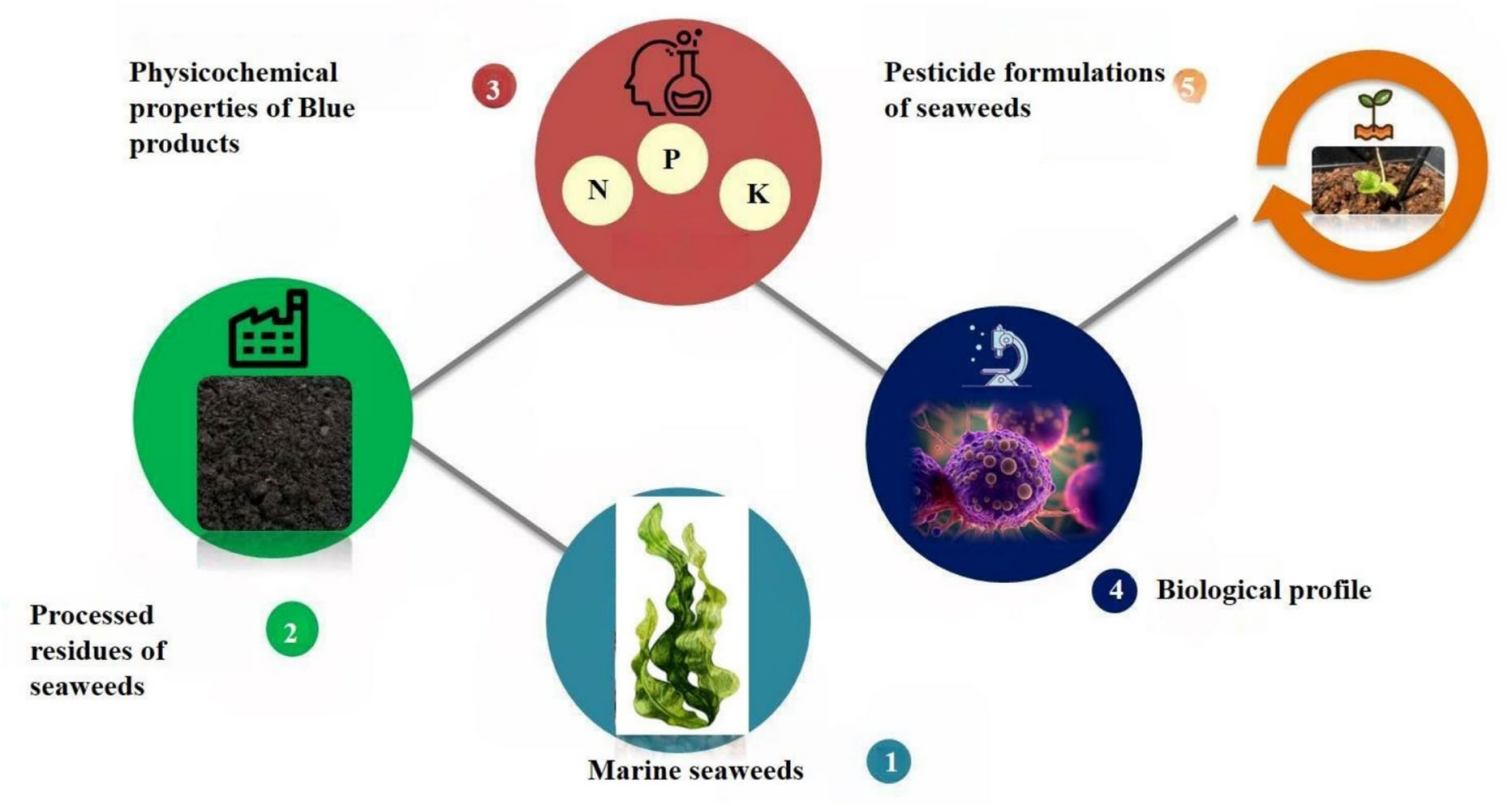
Biopesticides derived from seaweeds through different processes

The field of sustainable agriculture has shown a great deal of interest in seaweeds, especially with regard to their potential for producing Biopesticides (Fig. 3). Their significance arises from a range of distinct biochemical characteristics and environmental advantages, rendering them a compelling substitute for artificial pesticides. An in-depth examination of seaweeds' significance in the synthesis of biopesticides is provided below:

#### Rich source of bioactive compounds

A diversified genre of marine life, seaweeds are prolific in biologically active compounds exhibiting antiviral, antifungal, and antibacterial properties. Among these components are fatty acids, polysaccharides, pigments, and phenolic substances. These substances are extracted and utilized to generate biopesticides, which are less destructive to non-target creatures than traditional pesticides and biodegradable. Research indicates that utilising seaweed extracts to build systemic resistance, plants can strengthen their innate defences against illness and pests (Keeffe et al. 2019). The benefits of seaweed are mostly due to its combination of minerals, vitamins, phenols, polysaccharides, sterols, and other bioactive ingredients. These compounds are thought to possess antioxidant, anti-inflammatory, anti-cancer, antibacterial, and anti-diabetic effects (El- Beltagi et al. 2022). Natural pigments (NPs) like carotenoids, chlorophylls, and phycobiliproteins are found in edible seaweed, and their advantageous medicinal qualities have made them well- known functional constituents (Manzoor et al. 2024).

Being the most prevalent type of macroseaweed, green seaweed is a valuable marine biological resource. It is abundant in a number of fatty acids, amino acids, dietary fibers, polysaccharides, polyphenols, pigments, and other active ingredients that are vital to many biological processes, including immunoregulation, antioxidant activity, and the anti-inflammatory response. The exploration and use of green seaweeds for increased economic value has intensified in recent years due to increased attention to marine resources (Xu et al. 2023). Green seaweed is a major source of several lipids and proteins. Additionally, seaweed contains lectins, glycoproteins, and phycobiliproteins (Table 1) (Echave et al. 2022).

**Table 1:** Seaweeds as rich source of bioactive compounds

Seaweed species	Biopesticide type	Potential bioactive compounds	References
*Ascophyllum* *nodosum*	Algicides	Fucoidans, polysaccharides, polyphenols	(Zaharudin et al. 2021)
*Fucus* *vesiculosus*	Herbicides	Phlorotannins, fucoidans, polyphenols	(Zandi et al. 2020)
*Gracilaria* *spp.*	Insecticides	Phycobiliproteins, polyphenols, sulfated polysaccharides	(Kumar et al. 2019)
*Ulva* *lactuca*	Fungicides	Lectins, sulfated polysaccharides, polyphenols	(Mishra et al. 2020)
*Sargassum* *spp.*	Nematicides	Peptides, alkaloids, polyphenols	(Zhou et al. 2024)
*Porphyra* *spp.*	Rodenticides	Peptides, alkaloids, polyphenols	(Ahn et al. 2021)
*Eisenia* *bicyclis*	Larvicides	Fucoidans, polyphenols, polysaccharides	(Li et al. 2018)
*Laminaria* *digitata*	Repellents	Phlorotannins, fucoxanthin, polyphenols	(Kim et al. 2019)

### Environmental sustainability

The use of seaweeds in biopesticide production aligns with the principles of environmental sustainability. Seaweeds grow abundantly in marine environments without the need for freshwater, arable land, or synthetic fertilizers, making them a low-impact resource. Moreover, seaweed cultivation can contribute to carbon sequestration and help mitigate the effects of eutrophication in marine ecosystems. By integrating seaweed-based products into agricultural practices, it is possible to reduce the ecological footprint of pest management strategies (Pereira and Cotas 2024). Using the natural substances found in seaweed, seaweed-based biopesticides help manage pests in agricultural settings while having fewer of an adverse effect on the environment than synthetic pesticides. The three aspects of seaweed's potential contributions to sustainability and environmental stewardship—carbon sequestration, mitigating ocean acidification, and applying biopesticides—represent distinct aspects of these contributions (Hurd et al. 2022). Since seaweed's capacity to absorb CO2 is essential to marine ecosystems and the worldwide cycling of carbon, its usage as biopesticides primarily targets food production and sustainable agriculture. This involves lowering the amount of chemical residues in food and the pollution that conventional pesticide use causes to the environment (Kaladharan et al. 2019).

### Safety and biocompatibility

Seaweed-based biopesticides are increasingly recognized as safe alternatives to chemical pesticides due to their natural origin, biodegradability, and minimal environmental impact. Seaweed-based biopesticides are also recognized as safe for humans and animals. This safety profile is particularly advantageous for crops used in human and animal foods, aligning with the global trend towards organic and clean-label products (Raja and Vidhya 2023).

### Economic benefits

The utilization of seaweeds in the manufacturing of biopesticides has significant economic ramifications. Seaweeds are a renewable resource that can be responsibly harvested to provide a steady supply of raw materials for the development of biopesticides. Compared to synthetic chemicals, which are frequently made from non-renewable petroleum sources, might be more affordable. Moreover, the increasing market for eco-friendly and organic products creates fresh prospects for seaweed-based biopesticides (Pereira and Cotas 2024).

### Innovation and research opportunities

Seaweeds offer vast potential for innovation in the field of agricultural biotechnology. The diverse chemical makeup of seaweeds presents opportunities for the discovery of novel bioactive compounds with unique modes of action against plant pathogens and pests. Ongoing research and development efforts are crucial to unlock these potentials, refine extraction and formulation techniques, and ensure the efficacy and stability of seaweed-based biopesticides (Ghaliaoui 2024). In conclusion, seaweeds hold a pivotal role in the future of biopesticides production, offering a sustainable, safe, and economically viable alternative to synthetic pesticides. Their integration into pest management systems represents a forward step towards more sustainable agricultural practices, highlighting the convergence of environmental stewardship and technological innovation.

## Overview of biopesticides

Natural, biologically based substances called biopesticides are applied to plants in forests, gardens, agricultural farms, and other areas to manage a variety of agricultural pests and they also helps to enhance crop production. Diverse biopesticide kinds have been developed from diverse sources (Samada & Tambunan 2020a, b; Kumar et al. 2021a, b). To improve the sustainability andefficacy of pest management, researchers are continuously looking for new sources of biopesticides, such as microbial strains, plant extracts, and natural chemicals.

### Seaweeds as a source of biopesticides

**Fig. 4:**
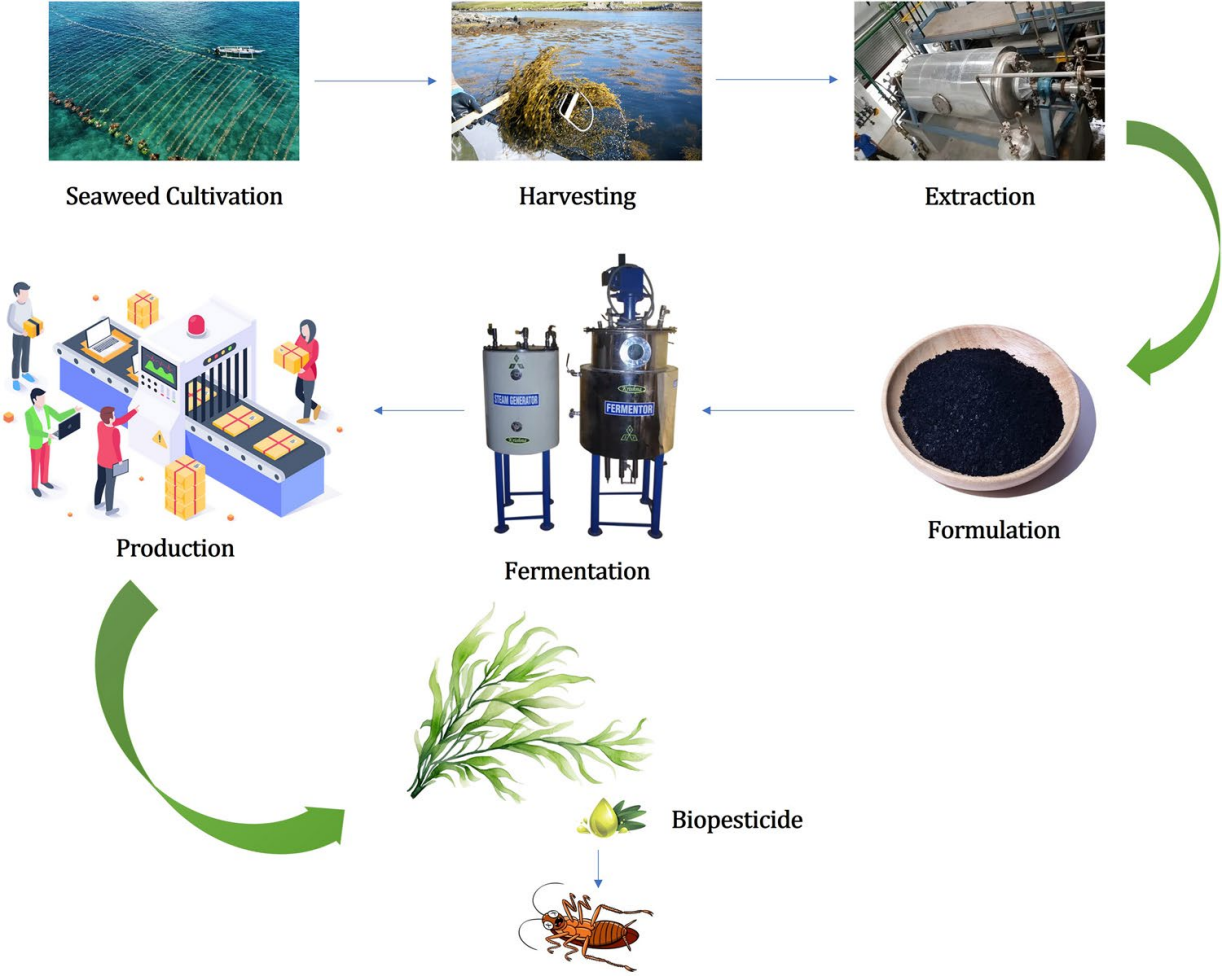
Production of organic pesticides from marine algae and usage in field land

Several studies have investigated the potential of various seaweeds in the production of biopesticides, focusing on their bioactive properties that can be utilized in controlling plant pathogens and enhancing agricultural sustainability (Fig. 4). Researchers reported 10,000 spices of marine macroalgae, known as seaweeds, are microscopic, multicellular, photosynthetic eukaryotic creatures Based on pigmentation, morphology, and anatomical characteristics, seaweeds are divided into three main groups: brown seaweeds (Phaeophyta), red seaweeds (Rhodophyta), and green seaweeds (Chlorophyta) (Guiry & Guiry 2017; El-Beltagi et al. 2022).

### Types of seaweeds with pest-repellent properties

#### Brown seaweeds (phaeophyta)

Brown seaweeds vary in dimension from small filamentous kinds to large/giant complex seaweeds, making them some of the largest and most complex marine algae (Wijesinghea 2012). Iodine and other special secondary metabolites, such phlorotannin, are abundant in marine algae. Researchers are giving these phenolic compounds a lot of attention because of their biological activity, which includes anti-viral, anti-cancer, cholesterol-lowering, and many other effects (Hakim and Patel 2020). Brown algae comprise primarily of polysaccharides with variety of unique physical and chemical properties due to their diverse structures. These properties include immunomodulation, antibacterial, antioxidant, prebiotic, antihypertensive, antidiabetic, antitumor, and anticoagulant activities, among many more, and help to moderate a wide range of biological activities. Alginate, laminarin, and fucoidan are the three main types of polysaccharides found in brown algae (Li et al. 2021). The potency of brown seaweeds, such as Ascophyllum nodosum and Sargassum species, in enhancing plant resistance to pests and diseases has been the object of much research. These seaweed extracts have the ability to give plants systemic acquired resistance, which reduces their vulnerability to insect and microbial pathogen attacks (Shukla et al. 2021).

#### Red seaweeds (rhodophyta)

Red seaweeds are the largest group of seaweeds, including around 6000 different species. They are known for having a high concentration of potential molecules like vitamins, minerals, phycobiliproteins, essential fatty acids, and other secondary metabolites, as well as bioactive sulphated polysaccharides like agar and carrageenan (Carpena 2022). Because of their antibacterial and antifungal properties, species including Porphyra and Laurencia have demonstrated promise in the treatment of pests. Specifically, the carrageenan found in red seaweeds have the ability to impede the growth of fungi, which is advantageous for managing fungal diseases in agricultural settings. Furthermore, certain red seaweeds have been utilized to create nematicides that effectively combat nematodes that harm a variety of crops (Mickymaray and Alturki 2018).

#### Green seaweeds (chlorophyta)

The intertidal zone is inhabited chiefly to green seaweeds. Common green seaweed species are found in the genera Ulva, Enteromorpha, Chaetomorpha, Codium, and Caulerpa. Ulva lactuca, U. prolifera, and U. linza are the four significant green seaweed species in the genus Ulva (Miao et al. 2020). Green seaweeds, like their brown and red counterparts, contain a variety of bioactive compounds, including peptides, fatty acids and phenolics, which exhibit biological activities against pests (Lomartire and Gonclaves 2022). Monostroma, Ulva and Caulerpa are commercially important seaweeds. Especially, Ulva (also known as sea lettuce) have been researched for their capacity to produce substances that repel insects and inhibit the growth of harmful bacteria and fungi. Green seaweeds are often easier to process and extract, making them an attractive option for biopesticide formulation (Xu et al. 2023).

#### Integration into agricultural practices

The integration of seaweed-based biopesticides into agricultural practices involves the formulation of these extracts into usable products such as sprays, powders, or integrated pest management systems. Seaweed extracts promote early seed germination and establishment, boost root growth, increase leaf chlorophyll, improve crop performance and elevate resistance to biotic/abiotic stress (Yao 2020).

#### Future research directions

Ongoing research is crucial to fully understand the spectrum of bioactive compounds in seaweeds and their mechanisms of action against various agricultural pests. Future studies are needed to standardize extraction methods, improve the stability and efficacy of seaweed-based biopesticides, and ensure their compatibility with other pest management strategies. Additionally, exploring the synergistic effects of combining different types of seaweed extracts could lead to the development of broad-spectrum biopesticides that are more effective and environmentally benign. In conclusion, seaweeds as a source of biopesticides represent a promising area of biotechnological innovation in agriculture, leveraging the natural pest-repellent properties of marine algae to develop safer, more sustainable pest management solutions.

### Bioactive compounds in seaweeds

**Fig. 5:**
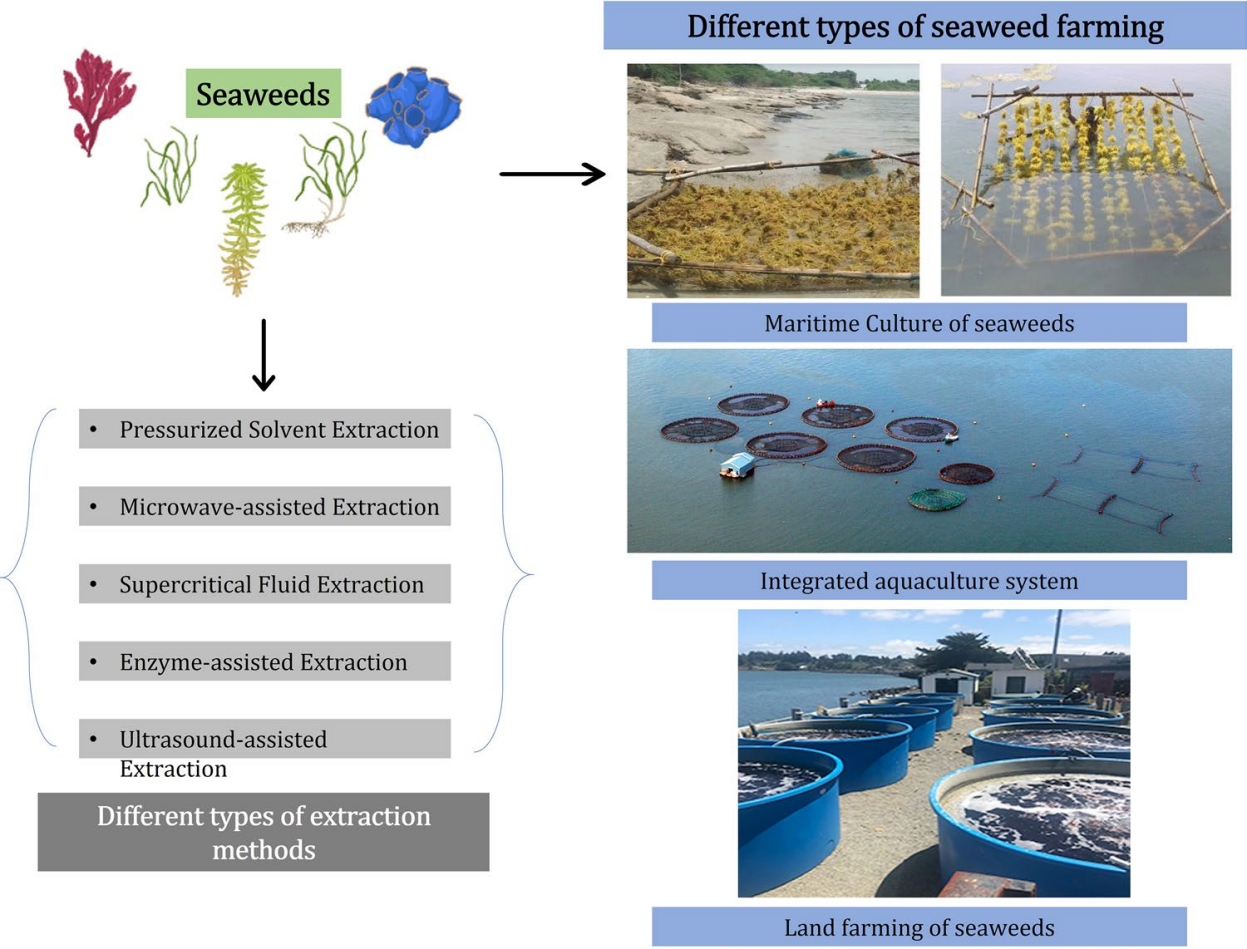
Cultivation, harvesting and bioprocessing of pesticides derived from seaweeds through different processes

Seaweeds contain elaborate secondary metabolites that play a significant role in the defence of the host against predators and parasites which offer a potential novel approach to control population of plant parasitic nematodes (JACOBS 2003). Seaweeds are an abundant source of bioactive compounds that hold potential for various applications, including the development of natural products such as biopesticides (Fig. 5). The bioactive compounds such as terpenoids, polyphenols, and sulfated polysaccharides, in particular, have been extensively studied for their biological properties. Here is an elaboration on these key compounds found in seaweeds.

#### Terpenoids

Terpenoids, also known as isoprenoids, are a large group of organic chemicals that are found in nature. They are made from five-carbon isoprene units that are arranged in a variety of ways. Terpenoids in seaweeds are effective natural pesticides due to their strong antifungal and antibacterial properties. They protect seaweeds from microbial invasions and herbivorous marine animals in crucial ways. Terpenoids, for instance, are produced by certain species of brown and red seaweed that repel marine herbivores and prevent fouling organisms from colonizing the area. Biopesticides that similarly protect agricultural crops from pests and diseases can be developed using these properties. Terpene based biopesticides show promise against aphid pests of ornamental crops (Smith 2018).

#### Polyphenols

Another significant class of compounds found in seaweeds are polyphenols, which are well-known for their antioxidant properties. The polyphenolic compounds such as phlorotannins, bromophenols, flavonoids, phenolic terpenoids, and mycosporine-like amino acids. In the brown seaweeds, the phlorotannins are the major polyphenolic class found only in the marine brown seaweeds. On the other hand, the largest proportion of phenolic compounds present in green and red seaweeds are bromophenols, flavonoids, phenolics acids, phenolic terpenoids (Wells et al. 2017; Corona et al. 2017; Gómez-Guzmán et al. 2018). Phlorotannins, a type of tannin found in earthy colored green growth, are one example of polyphenols from ocean growth that exhibit strong antibacterial and antifungal properties when it comes to biopesticides. These substances have the ability to stop the growth of several plant pathogens, which lowers the incidence of agricultural diseases. Moreover, polyphenols have the ability to stimulate defense mechanisms in plants, enhancing their resistance to attacks by microbes and rodents (Zheng et al. 2022).

#### Sulphated polysaccharides

Sulfated polysaccharides are unique to marine environments and are present in a wide range of seaweeds. These substances include fucoidan, ulvan, and carrageenan, which are found in red, brown, and green seaweeds, respectively. It is well known that sulfated polysaccharides have antibacterial, antifungal, and antiviral properties. They can directly prevent the growth of bacteria or cause plants to develop resistance. For example, fucoidans have been demonstrated to strengthen plants' defenses against fungus and viruses. Due to these characteristics, sulfated polysaccharides are crucial components of biopesticides, enhancing their viability and range of action (Liyanage et al. 2023).

The application of these bioactive compounds in agriculture as biopesticides offers a sustainable alternative to synthetic chemicals. Furthermore, the use of such natural products is often more favorable under organic farming regulations, enhancing the appeal and marketability of the produce (Ali et al. 2021). Crop performance may be impacted by the use of seaweed extracts and their bioactive components. Seaweed products provide many benefits for agriculture, including improved nutrient uptake, improved morphological and physiological traits, and reduced stress in plants. They have previously been shown to function in plants as both biostimulants and bioelicitors. Additionally, the Seaweed Extracts have no adverse effects on the environment and effectively stimulate plants' natural defenses. To maximize output and minimize the need for chemical fertilizers, Seaweed Extracts can be used in conjunction with other biofertilizers (Raja and Vidhya 2023).

While the potential of these bioactive compounds is significant, ongoing research is necessary to optimize their extraction, stability, and formulation for agricultural use. Advanced techniques in biotechnology and nanotechnology could play pivotal roles in enhancing the efficacy and delivery systems of seaweed-based biopesticides. Moreover, understanding the synergistic effects between different bioactive compounds could lead to the development of more effective and broad-spectrum biopesticides. The genomes of a variety of plants have now been sequenced or are close to being sequenced, researchers need to investigate the effects of seaweed extracts and components on the whole genome/transcriptome of plants to better understand the mechanisms of action of seaweed-induced growth response and stress alleviation (Poddar 2021). In conclusion, the bioactive compounds in seaweeds offer promising prospects for the development of natural and effective biopesticides. These compounds not only help in managing pests and diseases but also contribute to sustainable agricultural practices, which are crucial for the long-term health of ecosystems and human populations.

## Extraction methods

Bioactive components from seaweeds must be extracted in order to properly utilize them for a variety of purposes, including the production of biopesticides. Different methods are employed to ensure the effective and successful recovery of these materials; each method has unique benefits and applications (Table 2).

**Table 2:** Types of bioactive compounds extraction methods from seaweeds

Seaweed species	Biopesticide type	Extraction method	Mechanism	References
*Ascophyllum* *nodosum*	Algicides	Supercritical Fluid Extraction	Extraction of sulfated polysaccharides, such as fucoidan, known for their algicidal activities.	(Choi et al. 2017)
*Fucus* *vesiculosus*	Herbicides	Solvent Extraction	Extraction of bioactive compounds with herbicidal properties, such as polyphenols and alkaloids.	(Kumar et al. 2020)
*Gracilaria* *spp.*	Insecticides	Microwave- Assisted Extraction	Extraction of bioactive compounds, including phycobiliproteins and polyphenols, with insecticidal and larvicidal effects.	(Kumar et al. 2019)
*Ulva* *lactuca*	Fungicides	Aqueous Extraction	Extraction of bioactive compounds with antifungal properties, such as sulfated polysaccharides and lectins.	(Patra et al. 2018)
*Sargassum* *spp.*	Nematicides	Enzyme- Assisted Extraction	Release of bioactive peptides with nematicidal properties, disrupting the physiological processes of nematode pests.	(Yang et al. 2019)
*Porphyra* *spp.*	Rodenticides	Acid Hydrolysis	Hydrolysis of seaweed carbohydrates into bioactive compounds with rodenticidal effects, such as peptides and alkaloids.	(Lin et al. 2016)
*Eisenia* *bicyclis*	Larvicides	Enzymatic Hydrolysis	Enzymatic breakdown of seaweed polysaccharides into fermentable sugars, fermenting into larvicidal compounds through microbial processes.	(Yang et al. 2021)
*Laminaria* *digitata*	Repellents	Hydrothermal Liquefaction	Conversion of seaweed biomass into bio- oil through high-temperature and high- pressure conditions, producing compounds with repellent properties.	(Fang et al. 2020)

### Aqueous extraction

Aqueous extraction is one of the simplest and most environmentally friendly methods for extracting bioactive compounds from seaweeds. This method involves using water as the solvent, which is particularly effective for extracting hydrophilic compounds such as polysaccharides and certain proteins and peptides. Aqueous extraction is favored for its safety, low cost, and minimal environmental impact. It is also suitable for compounds that are heat-sensitive, as this method can be conducted at controlled temperatures to prevent degradation of the bioactive materials (Quitério 2022). Aqueous extraction is preferred for its simplicity, sustainability, and ability to preserve the natural integrity of seaweed-derived compounds. The efficacy of aqueous extraction methods in obtaining high yields of bioactive compounds from seaweed, including polysaccharides (e.g., agar, carrageenan), polyphenols, and peptides (Maskur et al. 2023).

### Supercritical fluid extraction (SFE)

A more sophisticated and effective technique for removing lipophilic chemicals from seaweeds is supercritical fluid extraction, especially when carbon dioxide (CO2) is used. SFE functions at solvent conditions above its critical point, where it displays special characteristics that set it apart from liquids and increase its solvation power. For the extraction of terpenoids, fatty acids, and other non-polar substances, this technique is quite successful. In addition to being eco- friendly and leaving no solvent residues behind, SFE has low working temperatures, great selectivity, and helps maintain the integrity of thermolabile molecules. Moreover, SFE's scalability makes it appropriate for industrial applications where environmental concerns and great efficiency are critical (Uwineza and Waśkiewicz 2020). The use of sub- and supercritical fluids for extraction is one of these technologies that is gaining more and more attention. By using these extraction technologies, the problems of selectivity and toxicity related to the use of organic solvents can be minimized. Pigments and other lipid-soluble substances can be selectively extracted through the use of supercritical carbon dioxide extraction (Getachew et al. 2020). Seaweed bioactive compounds can be extracted using energy-efficient and selective extraction techniques. Supercritical fluid extraction (SFE) techniques were widely used by companies to extract important bioactive chemicals from seaweeds. At different temperatures, pressures, and co-solvent percentage values, the seaweed extracted using the supercritical fluid extraction method has the highest level of antioxidant activity due to its largest concentration of total phenols (Ruiz- Domínguez 2022). Supercritical fluid extraction offers an acceptable method for the extraction of bioactive chemicals from seaweed, providing benefits over conventional extraction techniques in the areas of purity, selectivity, and environmental sustainability (Makur et al. 2023).

### Microwave-assisted extraction (MAE)

Microwave-Assisted Extraction uses microwave energy to heat the solvent and plant material, enhancing the extraction process's speed and efficiency. This method is particularly useful for extracting a wide range of bioactive compounds from seaweeds, including polyphenols and sulphated polysaccharides. MAE reduces the extraction time significantly, requires less solvent, and can result in higher yields compared to traditional methods. It is also noted for its ability to disrupt cell walls, facilitating the release of bioactive compounds. The rapid heating produced by microwaves leads to increased penetration of the solvent into the biomass, resulting in faster and more complete extraction (Makur et al. 2023).

### Ultrasound-assisted extraction (UAE)

Ultrasound refers to sound waves beyond the audible frequency range (typically, > 20 kHz). UAE method has been utilized in extraction of bioactive compounds from several marine algae or even non-hydrocolloids materials (Kumar et al. 2021a, b). A study on the extraction of carrageenan from *Hypnea musciformis*, a red seaweed species and found under the same circumstances, he discovered that employing UAE-alkaline and aqueous treatment techniques produced noticeably more carrageenan yields than the traditional approach. Interestingly, nuclear magnetic resonance (NMR) and Fourier-transform infrared spectroscopy (FTIR) methods indicated that the carrageenan obtained with these methods was highly purified and equivalent to conventional commercial carrageenan. Furthermore, the glucose polysaccharide laminarin was effectively isolated by other researchers from the Irish brown seaweed species Laminaria hyperborean and Ascophyllum nodosum. When compared to the traditional solid–liquid extraction approach, they found that for both brown seaweed species, the UAE method with HCl alteration showed the maximum extraction yield and antioxidant activity (Kadam et al. 2015a, b; Rafiquzzaman et al. 2016). Ultrasound-Assisted Extraction method has been extensively used in recent years in order to extract diverse BCs from an extensive spectrum of algae, especially polysaccharides like alginate and carrageenans; pigments like fucoxanthin, chlorophylls, or β-carotene; and phenolic compounds, among others. Utilizing Ultrasound-Assisted Extraction to marine algae is therefore a productive and environmentally friendly method of further exploring their profound characterization as a novel supply of BCs, especially appropriate for vegan and vegetarian diets (Carreira-Casais et al. 2021).

### Enzyme-assisted extraction (EAE)

Proteins, various binding ions like calcium and magnesium, and branching and sulphated polysaccharides make up the cuticles and cell walls of seaweed. The targeted compounds cannot be recovered from seaweed due to the physical barrier this chemical composition creates (Bhuyan et al. 2023). Therefore, in order to isolate the desired molecules, it is important to break downthese complex cell walls (Kulshreshtha et al. 2015; Grosso et al. 2015; Oliveira et al. 2020). The EAE method offers several advantages, including its eco-friendliness and low cost. A large yield of bioactive compounds can be produced by this technique, which can change molecules that are insoluble in water into ones that are soluble (Kadam 2013; Grosse 2015; Awad et al. 2021). Furthermore, EAE increases the antioxidant and antiviral effects of bioactive substances while also dramatically increasing their extraction yield. Unless they are tuned, standard extraction techniques usually do not yield this kind of result (Ghendov-Mosanu et al. 2022).

Each extraction method has its specific implications for the commercial production of biopesticides from seaweed. For instance, aqueous extraction is highly suitable for products that require a "natural" label, while SFE and MAE provide advantages in terms of efficiency and environmental sustainability. Ongoing advancements in extraction technologies may further enhance the effectiveness and reduce the costs of these methods, promoting their wider application in the industry. Allow high yield of bioactive compounds and Improve extraction yields and its quality.Optimizing the extraction of bioactive compounds from seaweeds is essential for maximizing their potential in various applications, including biopesticides. The choice of extraction method depends on the specific compounds of interest, the intended use, and the scale of production. Continued research and development in this area are crucial for improving the sustainability and efficacy of these natural products in agricultural settings.

## Challenges and limitations

### Optimization of extraction processes

With a methodical approach and the use of contemporary technologies, seaweed extraction procedures are able to rendered much more productive, sustainable, and efficient. Every stage needs to be precisely customized to the unique qualities of the seaweed species and the final goods that are intended (Pereira and Cotas 2023; Maskur et al. 2024). It is critical to take into account the technical benefits of each extraction technique as well as its consequences for product quality and economic feasibility when optimizing extraction methods for the commercial manufacture of biopesticides from seaweed (Maskur et al. 2024). Higher temperatures result in low yield of extraction and lower temperatures result in high yield of bioactive compounds. To optimize yield without compromising product integrity, alter the temperature and intensity of the extraction process (Chouaibi 2022). Adjust pH levels to optimize the effects of a specific seaweed bioactive components. To improve extraction efficiency, optimize the size of the seaweed particles (Perez- Vazquez et al. 2023). Utilize co-solvents to enhance the extraction yields and solubility of particular bioactive compounds. Certain beneficial compounds can be selectively extracted from seaweed (Uwineza and Waśkiewicz 2020). With microwave-assisted extraction techniques to achieve optimal extraction results, choose solvents that are compatible with microwave energy.

Modify power levels and extraction periods to improve quality and yield. It is important to control and monitor temperature to prevent the degradation of sensitive bioactive compounds. (Zhang et al. 2018).

### Formulation and stability of biopesticides

In biopesticides, organisms like bacteria, fungi, or viruses are often encompassed in formula. Because of the intrinsic diversity of these organisms, it might be challenging to guarantee consistency in biological activity and efficacy across batches (Damalas and Koutroubas 2018). Formulations known as biopesticides are made from substances that occur naturally like bacteria, plants, animals, and minerals. Their ability to effectively manage pests while reducing their negative effects on the environment makes them essential to sustainable agriculture (Kumar et al. 2021a, b). Identifying effective active ingredients from natural sources can be challenging. To identify compounds that are effective against pests and safe for the environment, a great deal of research and testing is necessary (Pathak et al. 2022). Integrating active ingredients with inert materials (solvents, adjuvants, and carriers) while maintaining stability and effectiveness is challenging. Unfavorable component interactions or gradual ingredient degradation may affect the biopesticide's effectiveness (Freeman et al. 2022). The sensitivity of biopesticides to environmental factors like humidity, light, and temperature is ubiquitous. To keep them effective until they are ready to use, stability during storage and transit is essential (Abbaszadeh et al. 2011; Kumar et al. 2021a, b). Biopesticides, as an alternative to synthetic pesticides, degrade faster in the field as a result of microbial activity, UV exposure, or other environmental conditions. It is difficult to make sure they endure long enough to efficiently manage pests without endangering beneficial creatures (Ayilara et al. 2023). Formulations for biopesticides need to be safe for users, non-target organisms, and applicators alike. Admixtures and carriers must be thoroughly tested and adhere to regulatory criteria in order to guarantee that they do not pose health concerns or harm the environment (Kumar et al. 2021a, b).

### Cost-effectiveness of production

Particularly contrasted to conventional chemical pesticides, the production of seaweed biopesticides can provide a number of economic benefits. Seaweed is often harvested ethically and is common in coastal areas. Compared to synthetic chemicals, which need more involved manufacturing procedures, this lowers the cost of raw materials. Seaweeds mostly rely on sunshine and the nutrients found in seawater for growth, requiring very little in the way of external inputs. When compared to terrestrial crops, this results in lower cultivation costs (Pereira and Cotas 2024). Costs associated with environmental compliance and cleanup may decrease as a result (Ayilara et al. 2024). Many coastal regions have suitable conditions for seaweed cultivation, allowing for local production and reducing transportation costs associated with importing chemical pesticides (UNO 2024). Numerous bioactive substances found in seaweed, including fatty acids, polysaccharides, and polyphenols, may have pesticidal effects. It may be possible to avoid expensive manufacturing procedures by extracting these chemicals and using them straight away as biopesticides (Lomartire and Gonçalves 2022). Incorporating seaweed biopesticides into an integrated pest control plan might reduce the need for expensive chemical treatments while encouraging environmentally friendly farming methods (Ali et al. 2021). Demand from consumers for environmentally friendly and sustainable agricultural goods is rising. By entering this market, seaweed biopesticides may be able to fetch higher prices and cover their production expenses (Upadhyay et al. 2020). Cost-effectiveness of seaweed biopesticides can vary based on factors such as local environmental conditions, regulatory requirements, scale of production, and technological advancements in extraction and formulation techniques (Pereira and Cotas 2024).

### Market demand and pricing

Concern regarding the effects of chemical pesticides on the environment is growing on a global scale. Sustainable and environmentally friendly solutions are what farmers and consumers are looking for. Concerns about the environment and human health are causing governments and regulatory agencies in many countries to tighten prohibitions on the use of synthetic pesticides. As a result, the regulatory landscape is more favorable to the use of biopesticides, particularly those made from seaweed (UNO 2024). For seaweed biopesticides to be recognized in the market, their efficacy against the intended pests is essential. The evidence from studies and field tests showing their superiority over conventional pesticides is a major factor in demand. Because synthetic chemicals are forbidden in organic farming systems, seaweed biopesticides are especially desirable. The market for biopesticides is robust and expanding in this area of agriculture (Kumar et al. 2021a, b). Creative approaches to pest management are required as agriculture diversifies to satisfy shifting consumer needs and adjust to climate change. In this sense, seaweed biopesticides present a special opportunity (Helseena et al. 2023). A number of variables, including the type of seaweed utilized, the cultivation and extraction procedures, and the formulation methods, might affect the cost of making seaweed biopesticides. Biopesticides may be more cost-effective than conventional pesticides due to lower production costs (Chukwuma et al. 2024). Because of their perceived safety and eco-friendliness, biopesticide including those made from seaweed often fetch higher costs than synthetic pesticides. Positioning as premium items within the organic and sustainable agriculture market segments is one possible strategy for pricing (Fenibo et al. 2021). Producers financial soundness must be taken into account while setting prices. Biopesticides can provide long-term advantages including better soil health and less environmental impact, even if they may have greater upfront costs or lower application rates than chemical pesticides (UNO 2024). Seaweed pesticides are in high demand due to their effectiveness in controlling pests, sustainability concerns, and regulatory demands. In order to effectively seize market opportunities in the always changing agricultural landscape, pricing strategies ought to strike a balance between production costs, market positioning, and competitive dynamics (Pereira and Cotas 2024).

### Registration and approval processes

In the EU, medicinal products containing seaweed components must abide by EU medicine law. The EU's guidelines for human medications are outlined in Directive 2001/83/EC. According to Directive 2001/83/EC, Title I, Article 1, "(a) Any substance or combination of substances presented as having properties for treating or preventing disease in humans; or (b) Any substance or combination of substances which may be used in or administered to humans either with a view to making a medical diagnosis, or to restore, correct, or modify physiological functions by exerting a pharmacological, immunological, or metabolic action." The EU Commission issues the license through a centralized mechanism after the European Medicines Agency assesses the evidence. The processes for mutual recognition and national recognition (Lähteenmäki-Uutela 2021). Seaweed biopesticide registration and approval procedures involve meticulous preparation, adherence to legal requirements, and solid scientific evidence to support safety and efficacy. Smoother regulatory approval and market entry can be facilitated by consulting with regulatory authorities early in the development process and seeking professional advice (Chandler et al. 2011).

### Compliance with safety and environmental regulations

To evaluate the possible health risks associated with the seaweed pesticides, carry out thorough toxicology investigations. Studies on skin sensitization, ocular irritation, dermal toxicity, inhalation toxicity, and acute toxicity are included in this. Assess possible human exposure during handling, application, and post-application scenarios via different channels (oral, dermal, and inhalation). Determining safe handling practices and application rates is aided by this assessment. To ascertain whether residues are present and at what levels in food and environmental matrices following application, conduct residue studies. Maximum residual limits (MRLs) should be established to guarantee food safety and adherence to legal requirements (Adams et al. 2024). Undertake research to evaluate the behavior of pesticides derived from seaweed in the environment, taking into account factors such as degradation rates, persistence, mobility, and bioaccumulation potential. This data is essential for evaluating environmental hazards. Assess the possible negative consequences of seaweed pesticides on non-target organisms, including beneficial insects, aquatic life, and soil microbes. Studies on acute and chronic toxicity are frequently included in assessments. To do a risk assessment, use the information gathered from environmental destiny and ecotoxicity studies. Assess possible threats to non-target species and ecosystems, and if necessary, suggest mitigation strategies (Sundhar et al. 2020).

## Future perspectives

### Potential for seaweeds in biopesticides market

Seaweeds show great promise as environmentally friendly substitutes for conventional chemical pesticides, according to research on their potential in the biopesticide industry. Seaweeds, also known as macroalgae, are rich in bioactive substances with pesticidal qualities, including polysaccharides, polyphenols, and halogenated chemicals. These substances can function as chemicals, growth inhibitors, or repellents against infections and pests. The biopesticide industry is now beginning to understand the potential of seaweeds because of their natural components that reduce pests and improve the environment (Duarte et al. 2021). Numerous bioactive substances, including polysaccharides (such as alginates and carrageenans), polyphenols, and terpenes, are found in seaweeds. These substances have demonstrated pesticidal effects on nematodes, fungi, and insects (Hentali 2020). An invasive seaweed that may be used as a biopesticide is Asparagopsis armata. Its exudate, when given to a model weed, has detrimental effects on energy metabolism and carotenoid metabolism, and it also dramatically increases oxidative stress. This implies that this macroalga could be used in a biopesticide cocktail, which is an environmentally friendly and long-lasting way to manage weeds.

### Innovation in biopesticide technology

Nanoformulation improve the active components' targeted delivery, stability, and bioavailability (Afzal et al. 2022). Utilizing nanotechnology, biologically synthesized pesticides were created from inexpensive sources like seaweed, particularly green algae, since it is safe to create pesticides against a variety of pests that harm different types of crops, including fungi and bacteria (Amin 2020). Microencapsulation helps to preserve bioactive substances, extend their release, and boost effectiveness in a range of environmental circumstances. Alginate is a biopolymer that is frequently used for this kind of microencapsulation; it is derived from seaweed. Alginate-based microcapsules that satisfy essential bacterial encapsulation criteria, such as biocompatibility and biodegradability, and that sustain long-term survival and function, have been produced as a consequence of recent advancements (Saberi Riseh et al. 2021).

### Importance of sustainable practices and environmental protection

In recent years, there has been a renewed focus on identifying sustainable alternatives to conventional chemical pesticides due to concerns about pesticide resistance, hazards to human health, and the environment. A possible solution has developed in the form of seaweed biopesticides, which effectively control pests by using natural chemicals derived from marine algae (Ayilara et al. 2023).

## Conclusion

Marine biomass forms the largest component of marine ecosystems and offers numerous benefits, including nutrition, agar, algin, carrageenan production, and biofuels. Its use as a biopesticide source holds great potential for sustainable agriculture and environmental conservation. Bioactive compounds in marine organisms like seaweed, microalgae, and marine bacteria can efficiently control pests with minimal impact on ecosystems and non-target organisms. The diversity of marine biomass enables the development of tailored biopesticides for specific agricultural challenges, reducing reliance on synthetic pesticides and supporting biodiversity conservation. Despite this potential, challenges remain in scaling production, optimizing extraction, and ensuring cost competitiveness. Advanced formulation techniques like nanoencapsulation and microemulsions can improve stability and targeted delivery of bioactive compounds (Pateiro et al. 2021). Further research is essential to understand the environmental fate of seaweed biopesticides and their ecosystem impacts. Streamlined regulatory frameworks and standards are necessary to ensure market access and product efficacy (Samada et al. 2020). Collaboration among governments, industries, academia, and farmers can accelerate the development and adoption of seaweed-based biopesticides. Investments in seaweed cultivation, extraction, and production infrastructure, coupled with farmer education, can enhance their sustainable use (Mishra et al. 2020). With innovative approaches and policy support, seaweed biopesticides can significantly contribute to resilient crops, ecosystem preservation, and agricultural sustainability.

## Data Availability

Not applicable.

## References

[CR1] Abbaszadeh G, Dhillon M, Srivastava C, Gautam R (2011) Effect of climatic factors on bioefficacy of biopesticides in insect pest management. Biopesticides Int 7:1–14

[CR2] Abdullah LL, Javed HU, Xiao J (2022) Engineering emulsion gels as functional colloids emphasizing food applications: a review. Front Nutr 9:890188. 10.3389/fnut.2022.890188 35656162 10.3389/fnut.2022.890188 PMC9152362

[CR3] Achmad H, Huldani H, Feby Ramadhany Y (2020) Antimicrobial activity and sulfated polysaccharides antibiofilms in marine algae against dental plaque bacteria: a literature review. Syst Rev Pharm 11:459–465

[CR4] Ádám B, Cocco P, Godderis L (2024) Hazardous effects of pesticides on human health. Toxics 12(3):186. 10.3390/toxics12030186 38535918 10.3390/toxics12030186 PMC10975530

[CR5] Afzal O, Altamimi ASA, Nadeem MS, Alzarea SI, Almalki WH, Tariq A, Mubeen B, Murtaza BN, Iftikhar S, Riaz N, Kazmi I (2022) Nanoparticles in drug delivery: from history to therapeutic applications. Nanomaterials 12(24):4494. 10.3390/nano12244494 36558344 10.3390/nano12244494 PMC9781272

[CR121] Ahn J, Kim MJ, Ahn J, Ha TY, Jung CH, Seo HD, Jang YJ (2021) Identifying Codium fragile extract components and their effects on muscle weight and exercise endurance. Food Chem 353:129463 33743428 10.1016/j.foodchem.2021.129463

[CR6] Ali O, Ramsubhag A, Jayaraman J (2021) Biostimulant properties of seaweed extracts in plants: implications towards sustainable crop production. Plants 10(3):531. 10.3390/plants10030531 33808954 10.3390/plants10030531 PMC8000310

[CR7] Amin H (2020) Biosynthesized silver nanoparticles using *Ulva lactuca* as a safe synthetic pesticide (*in vitro*). Open Agric 2020(5):291–299

[CR8] Awad AM, Kumar P, Ismail-Fitry MR, Jusoh S, Ab Aziz MF, Sazili AQ (2021) Green extraction of bioactive compounds from plant biomass and their application in meat as natural antioxidant. Antioxidants 10(9):1465. 10.3390/antiox10091465 34573097 10.3390/antiox10091465 PMC8466011

[CR9] Ayilara MS, Adeleke BS, Akinola SA, Fayose CA, Adeyemi UT, Gbadegesin LA, Omole RK, Johnson RM, Uthman QO, Babalola OO (2023) Biopesticides as a promising alternative to synthetic pesticides: a case for microbial pesticides, phytopesticides, and nanobiopesticides. Front Microbiol 14:1040901 36876068 10.3389/fmicb.2023.1040901 PMC9978502

[CR106] Ayilara MS, Akinola SA, Adeleke BS, Gbadegesin LA, Adejumo GD, Glick BR, Babalola OO (2024) Biopesticides versus synthetic pesticides usage in Africa. In: Microbiome-based decontamination of environmental pollutants, pp 417–450

[CR10] Bhuyan PP, Nayak R, Patra S, Abdulabbas HS, Jena M, Pradhan B (2023) Seaweed-derived sulfated polysaccharides; the new age chemopreventives: a comprehensive review. Cancers 15(3):715. 10.3390/cancers15030715 36765670 10.3390/cancers15030715 PMC9913163

[CR95] Bibi R, Tariq RM, Rasheed M (2020) Toxic assessment, growth disrupting and neurotoxic effects of red seaweeds’ botanicals against the dengue vector mosquito Aedes aegypti L. Ecotoxicol Environ Saf 195:110451 32199214 10.1016/j.ecoenv.2020.110451

[CR11] Biris-Dorhoi ES, Michiu D, Pop CR, Rotar AM, Tofana M, Pop OL, Socaci SA, Farcas AC (2020) Macroseaweeds—a sustainable source of chemical compounds with biological activities. Nutrients 12(10):3085. 10.3390/nu12103085 33050561 10.3390/nu12103085 PMC7601163

[CR12] Bringloe TT, Sauermann R, Krause-Jensen D, Olesen B, Klimova A, Klochkova TA, Verbruggen H (2021) High-throughput sequencing of the kelp *Alaria* (*Phaeophyceae*) reveals epi-endobiotic associations, including a likely phaeophycean parasite. Eur J Phycol 56(4):494–504

[CR13] Cai J, Aguilar-Manjarrez J, Cornish L, Dabbadie L (2021) Seaweeds and microalgae: an overview for unlocking their potential in global aquaculture development. FAO Fisheries and Aquaculture Circular. FAO, Rome

[CR14] Cai J (2021a). Global seaweeds and microalgae production, 1950–2019.

[CR15] Carpena M, Garcia-Perez P, Garcia-Oliveira P, Chamorro F, Otero P, Lourenço-Lopes C, Cao H, Simal-Gandara J, Prieto MA (2022) Biological properties and potential of compounds extracted from red seaweeds. Phytochem Rev. 10.1007/s11101-022-09826-z 35791430 10.1007/s11101-022-09826-z PMC9247959

[CR16] Carreira-Casais A, Otero P, Garcia-Perez P, Garcia-Oliveira P, Pereira AG, Carpena M, Soria-Lopez A, Simal-Gandara J, Prieto MA (2021) Benefits and drawbacks of ultrasound-assisted extraction for the recovery of bioactive compounds from marine algae. Int J Environ Res Public Health 18(17):9153. 10.3390/ijerph18179153 34501743 10.3390/ijerph18179153 PMC8431298

[CR17] Chandler D, Bailey AS, Tatchell GM, Davidson G, Greaves J, Grant WP (2011) The development, regulation and use of biopesticides for integrated pest management. Philos Trans R Soc Lond B Biol Sci 366(1573):1987–1998. 10.1098/rstb.2010.0390 21624919 10.1098/rstb.2010.0390 PMC3130386

[CR18] Cherry P, O’Hara C, Magee PJ, McSorley EM, Allsopp PJ (2019) Risks and benefits of consuming edible seaweeds. Nutr Rev 77(5):307–329. 10.1093/nutrit/nuy066 30840077 10.1093/nutrit/nuy066 PMC6551690

[CR109] Choi S, Kang JW, Lee JH, Seong CN (2017) Dokdonia lutea sp. nov., isolated from Sargassum fulvellum seaweed. Int J Syst Evol Microbiol 67(11):4482–4486 28933321 10.1099/ijsem.0.002317

[CR19] Chouaibi M (2022) Supercritical carbon dioxide extraction of clove essential oil: optimization and characterization. Elsevier, Amsterdam, pp 531–540

[CR20] Chukwuma O, Tan SP, Hughes H, Mcloughlin P, O’Toole N, McCarthy N (2024) The Potential of seaweeds as a rich natural source for novel bioherbicide formulation/development. Weed Sci 72:1–30. 10.1017/wsc.2024.1

[CR99] Corona G, Coman MM, Guo Y, Hotchkiss S, Gill C, Yaqoob P et al (2017) Effect of simulated gastrointestinal digestion and fermentation on polyphenolic content and bioactivity of brown seaweed phlorotannin-rich extracts. Mol Nutr Food Res 61(11):1700223 10.1002/mnfr.201700223 28718977

[CR21] Corsetto PA, Montorfano G, Zava S, Colombo I, Ingadottir B, Jonsdottir R, Sveinsdottir K, Rizzo AM (2020) Characterization of antioxidant potential of seaweed extracts for enrichment of convenience food. Antioxidants 9(3):249. 10.3390/antiox9030249 32204441 10.3390/antiox9030249 PMC7139466

[CR22] Costa JAV, Freitas BCB, Cruz CG et al (2019) Potential of microalgae as biopesticides to contribute to sustainable agriculture and environmental development. J Environ Sci Health B 54:366–375. 10.1080/03601234.2019.1571366 30729858 10.1080/03601234.2019.1571366

[CR93] Cotas J, Lomartire S, Gonçalves AM, Pereira L (2024) From ocean to medicine: harnessing seaweed’s potential for drug development. Int J Mol Sci 25(2):797 38255871 10.3390/ijms25020797 PMC10815561

[CR23] Damalas CA, Koutroubas SD (2018) Current status and recent developments in biopesticide use. Agriculture 8:13. 10.3390/agriculture8010013

[CR24] Díaz-Tapia P, Nelson WA, Verbruggen H (2023) Molecular analyses of turf algae reveal a new species and an undetected introduction in the pterosiphonieae (rhodomelaceae, rhodophyta). J Phycol 59(3):603–618. 10.1111/jpy.13336 37070822 10.1111/jpy.13336

[CR25] Dixit D, Reddy C, Trivedi M, Gadhavi DK (2020) Non-targeted metabolomics approach to assess the brown marine macroalga *Dictyota dichotoma* as a functional food using liquid chromatography with mass spectrometry. Sep Sci plus 3:140–149

[CR26] Duarte B, Carreiras J, Feijão E, de Carvalho RC, Matos AR, Fonseca VF, Novais SC, Lemos MFL (2021) Potential of *Asparagopsis armata* as a Biopesticide for weed control under an invasive seaweed circular-economy framework. Biology 10(12):1321. 10.3390/biology10121321.PMID:34943236;PMCID:PMC8698409 34943236 10.3390/biology10121321 PMC8698409

[CR27] Echave J, Otero P, Garcia-Oliveira P, Munekata PE, Pateiro M, Lorenzo JM, Simal-Gandara J, Prieto MA (2022) Seaweed-derived proteins and peptides: promising marine bioactives. Antioxidants 11(1):176. 10.3390/antiox11010176 35052680 10.3390/antiox11010176 PMC8773382

[CR28] El-Beltagi HS, Mohamed AA, Mohamed HI, Ramadan KMA, Barqawi AA, Mansour AT (2022) Phytochemical and potential properties of seaweeds and their recent applications: a review. Mar Drugs 20(6):342. 10.3390/md20060342 35736145 10.3390/md20060342 PMC9227187

[CR116] Fang J, Fang J, Chen Q, Mao Y, Jiang Z, Du M et al (2020) Assessing the effects of oyster/kelp weight ratio on water column properties: an experimental IMTA study at Sanggou Bay, China. J Oceanol Limnol 38(6):1914–1924

[CR29] FAO (2023). Global fisheries and aquaculture at a glance. Available at https://www.fao.org/3/cc0461en/ online/sofia/2022/world-fisheries-aquaculture.html. FAO and WHO (2022). Report of the expert meeting on food safety for seaweed – Current status and future perspectives. Rome, 28–29 0ctober 2021. Food Safety and Quality Series No. 13. Rome.

[CR107] Fenibo EO, Ijoma GN, Matambo T (2021) Biopesticides in sustainable agriculture: A critical sustainable development driver governed by green chemistry principles. Front Sustain Food Syst 5:619058

[CR105] Freeman S, Greenfeld A, Angel D, Abbink W (2022) Deliverable 4.1: Salmon-seaweed coculture: beyond farm-level integrated multi-trophic aquaculture (IMTA): FutureEUAqua

[CR30] Getachew AT, Jacobsen C, Holdt SL (2020) Emerging technologies for the extraction of marine phenolics: opportunities and challenges. Mar Drugs 18:389. 10.3390/md18080389 32726930 10.3390/md18080389 PMC7459876

[CR31] Ghaliaoui N, Hazzit M, Mokrane H (2024) Seaweeds as a potential source of bioactive compounds. Res Biotechnol Environ Sci 3:1–8. 10.5880/rbes.v3i1.19

[CR102] Ghendov-Mosanu A, Cojocari D, Balan G, Patras A, Lung I, Soran ML et al (2022) Chemometric optimization of biologically active compounds extraction from grape marc: composition and antimicrobial activity. Molecules 27(5):1610 35268711 10.3390/molecules27051610 PMC8911792

[CR100] Gómez-Guzmán M, Rodríguez-Nogales A, Algieri F, Gálvez J (2018) Potential role of seaweed polyphenols in cardiovascular-associated disorders. Mar Drugs 16(8):250 30060542 10.3390/md16080250 PMC6117645

[CR88] Gomez-Zavaglia A, Prieto Lage MA, Jimenez-Lopez C, Mejuto JC, Simal-Gandara J (2019) The potential of seaweeds as a source of functional ingredients of prebiotic and antioxidant value. Antioxidants 8(9):406 31533320 10.3390/antiox8090406 PMC6770939

[CR32] Grosso C, Valentão P, Ferreres F, Andrade PB (2015) Alternative and efficient extraction methods for marine derived compounds. Mar Drugs 13:3182–3230. 10.3390/md13053182 26006714 10.3390/md13053182 PMC4446625

[CR33] Guiry MD, Guiry GM (2017) AlgaeBase. Worldwide electronic publication, National University of Ireland, Galway

[CR34] Hakim MM, Patel IC (2020) A review on phytoconstituents of marine brown algae. Futur J Pharm Sci 6:129. 10.1186/s43094-020-00147-6

[CR35] Helseena HE, Thampi M, Rebello S, Sheikhmoideen J (2023) Biopesticides: a green approach towards agricultural pests. Appl Biochem Biotechnol. 10.1007/s12010-023-04765-7 10.1007/s12010-023-04765-7 37994977

[CR36] Hentati F, Tounsi L, Djomdi D, Pierre G, Delattre C, Ursu AV, Fendri I, Abdelkafi S, Michaud P (2020) Bioactive polysaccharides from seaweeds. Molecules 25(14):3152. 10.3390/molecules25143152 32660153 10.3390/molecules25143152 PMC7397078

[CR37] Hurd C, Law C, Bach L, Britton D, Hovenden M, Paine E, Raven J, Tamsitt V, Boyd P (2022) Forensic carbon accounting: assessing the role of seaweeds for carbon sequestration. J Phycol. 10.1111/jpy.13249 35286717 10.1111/jpy.13249

[CR38] Jacobs H, Gray SN, Crump DH (2003) Interactions between nemato phagous fungi and consequences for their potential as biological agents for the control of potato cyst nematodes. Mycol Res 107:47–56 12735243 10.1017/s0953756202007098

[CR39] Kadam SU, Tiwari BK, O’Donnell CP (2013) Application of novel extraction technologies for bioactives from marine algae. J Agric Food Chem 61:4667–4675. 10.1021/jf400819p 23634989 10.1021/jf400819p

[CR40] Kadam SU, O’Donnell CP, Rai DK, Hossain MB, Burgess CM, Walsh D, Tiwari BK (2015a) Laminarin from Irish brown seaweeds *Ascophyllum nodosum**and Laminaria hyperborea*: ultrasound assisted extraction, characterization and bioactivity. Mar Drugs 13:4270–4280. 10.3390/md13074270 26184235 10.3390/md13074270 PMC4515616

[CR41] Kadam SU, Tiwari BK, Smyth TJ, O’Donnell CP (2015b) Optimization of ultrasound assisted extraction of bioactive components from brown seaweed *Ascophyllum nodosum* using response surface methodology. Ultrason Sonochem 23:308–316. 10.1016/j.ultsonch.2014.10.007 25453215 10.1016/j.ultsonch.2014.10.007

[CR42] Kaladharan P, Amalu AM, Revathy S (2019) Role of seaweeds in neutralizing the impact of seawater acidification- a laboratory study with beached shells of certain bivalves and spines of a sea urchin. J Mar Biol Ass India. 10.6024/jmbai.2019.61.1.2063-14

[CR123] Kim J, Stekoll M, Yarish C (2019) Opportunities, challenges and future directions of open-water seaweed aquaculture in the United States. Phycologia 58(5):446–461

[CR43] Kulshreshtha G, Burlot A-S, Marty C, Critchley A, Hafting J, Bedoux G, Bourgougnon N, Prithiviraj B (2015) Enzyme-assisted extraction of bioactive material from *Chondrus crispus* and *Codium fragile* and its effect on herpes simplex virus (HSV-1). Mar Drugs 13:558–580. 10.3390/md13010558 25603348 10.3390/md13010558 PMC4306952

[CR111] Kumar Y, Tarafdar A, Kumar D, Badgujar PC (2019) Effect of Indian brown seaweed Sargassum wightii as a functional ingredient on the phytochemical content and antioxidant activity of coffee beverage. J Food Sci Technol 56:4516–4525 31686683 10.1007/s13197-019-03943-y PMC6801239

[CR110] Kumar LR, Treesa Paul P, Anas KK, Tejpal CS, Chatterjee NS, Anupama TK et al (2020) Screening of effective solvents for obtaining antioxidant-rich seaweed extracts using principal component analysis. J Food Process Preserv 44(9):e14716

[CR44] Kumar K, Srivastav S, Sharanagat VS (2021a) Ultrasound assisted extraction (UAE) of bioactive compounds from fruit and vegetable processing by-products: a review. Ultrason Sonochem 70:105325. 10.1016/j.ultsonch.2020.105325 32920300 10.1016/j.ultsonch.2020.105325 PMC7786612

[CR45] Kumar J, Ramlal A, Mallick D, Mishra V (2021b) An overview of some biopesticides and their importance in plant protection for commercial acceptance. Plants 10(6):1185. 10.3390/plants10061185 34200860 10.3390/plants10061185 PMC8230470

[CR46] Lahlali R, Ezrari S, Radouane N, Kenfaoui J, Esmaeel Q, El Hamss H, Belabess Z, Barka EA (2022) Biological control of plant pathogens: a global perspective. Microorganisms 10(3):596. 10.3390/microorganisms10030596 35336171 10.3390/microorganisms10030596 PMC8951280

[CR47] Lähteenmäki-Uutela A, Rahikainen M, Camarena-Gómez MT, Piiparinen J, Spilling K, Yang B (2021) European union legislation on macroalgae products. Aquac Int 29:487–509. 10.1007/s10499-020-00633-x

[CR48] Levi PM, Norival S-F, Gasparoto MC (2019) Seaweeds in the control of plant diseases and insects. CRC Press, Boca Raton

[CR122] Li Y, Sun S, Pu X, Yang Y, Zhu F, Zhang S, Xu N (2018) Evaluation of antimicrobial activities of seaweed resources from Zhejiang Coast, China. Sustainability 10(7):2158

[CR49] Li Y, Zheng Y, Zhang Y, Yang Y, Wang P, Imre B, Wong ACY, Hsieh YSY, Wang D (2021) Brown algae carbohydrates: structures, pharmaceutical properties, and research challenges. Mar Drugs 19(11):620. 10.3390/md19110620 34822491 10.3390/md19110620 PMC8623139

[CR114] Lin HTV, Lu WJ, Tsai GJ, Chou CT, Hsiao HI, Hwang PA (2016) Enhanced anti-inflammatory activity of brown seaweed Laminaria japonica by fermentation using Bacillus subtilis. Process Biochem 51(12):1945–1953

[CR50] Liyanage NM, Nagahawatta DP, Jayawardena TU, Sanjeewa KKA, Jayawrdhana HHACK, Kim JI, Jeon YJ (2023) Sulfated polysaccharides from seaweeds: a promising strategy for combatting viral diseases-a review. Mar Drugs 21(9):461. 10.3390/md21090461 37755074 10.3390/md21090461 PMC10532895

[CR51] Lomartire S, Gonçalves AMM (2022) An overview of potential seaweed-derived bioactive compounds for pharmaceutical applications. Mar Drugs 20(2):141. 10.3390/md20020141 35200670 10.3390/md20020141 PMC8875101

[CR96] Lomartire S, Marques JC, Gonçalves AM (2022) An overview of the alternative use of seaweeds to produce safe and sustainable bio-packaging. Appl Sci 12(6):3123

[CR52] Manzoor MF, Afraz MT, Yılmaz BB, Adil M, Arshad N, Goksen G, Ali M, Zeng X-A (2024) Recent progress in natural seaweed pigments: Green extraction, health-promoting activities, techno-functional properties and role in intelligent food packaging. J Agric Food Res. 10.1016/j.jafr.2024.100991

[CR53] Maskur M, Mohammad S, Ketut S (2023) Environmental-friendly extraction methods to produce bioactive compounds in seaweed. Res J Chem Environ 27:114–121

[CR104] Maskur M, Sayuti M, Widyasari F, Setiarto RHB (2024) Bioactive compound and functional properties of sea cucumbers as nutraceutical products. Rev Agric Sci 12:45–64

[CR90] Mata L, Magnusson M, Paul NA, de Nys R (2016) The intensive land-based production of the green seaweeds Derbesia tenuissima and Ulva ohnoi: biomass and bioproducts. J Appl Phycol 28:365–375

[CR98] Miao X, Xiao J, Xu Q, Fan S, Wang Z, Wang X, Zhang X (2020) Distribution and species diversity of the floating green macroalgae and micro-propagules in the Subei Shoal, southwestern Yellow Sea. PeerJ 8:e10538 33362976 10.7717/peerj.10538 PMC7749999

[CR54] Mickymaray S, Alturaiki W (2018) Antifungal efficacy of marine macroalgae against fungal isolates from bronchial asthmatic cases. Molecules 23(11):3032. 10.3390/molecules23113032 30463364 10.3390/molecules23113032 PMC6278659

[CR55] Mishra A, Sahni S, Kumar S, Prasad B (2020) Seaweed -an eco- friendly alternative of agrochemicals in sustainable agriculture. Curr J Appl Sci Technol 39:71–78. 10.9734/CJAST/2020/v39i2730921

[CR92] Moon M, Park WK, Suh WI, Chang YK, Lee B (2019) Biological carbon recovery from sugar refinery washing water into microalgal DHA: medium optimization and stress induction. Sci Rep 9(1):19959 31882916 10.1038/s41598-019-56406-x PMC6934592

[CR97] O’Keeffe E, Hughes H, McLoughlin P, Tan SP, McCarthy N (2019) Antibacterial activity of seaweed extracts against plant pathogenic bacteria. J Bacteriol Mycol 6(3):1105

[CR101] Oliveira MC, Pellizzari F, Medeiros AS, Yokoya NS (2020) Diversity of Antarctic seaweeds. In: Antarctic seaweeds: diversity, adaptation and ecosystem services, pp 23–42

[CR56] Pacheco-Quito EM, Ruiz-Caro R, Veiga MD (2020) Carrageenan: drug delivery systems and other biomedical applications. Mar Drugs 18(11):583. 10.3390/md18110583 33238488 10.3390/md18110583 PMC7700686

[CR57] Pateiro M, Gómez B, Munekata PES, Barba FJ, Putnik P, Kovačević DB, Lorenzo JM (2021) Nanoencapsulation of promising bioactive compounds to improve their absorption, stability, functionality and the appearance of the final food products. Molecules 26(6):1547. 10.3390/molecules26061547 33799855 10.3390/molecules26061547 PMC7999092

[CR58] Pathak VM, Verma VK, Rawat BS, Kaur B, Babu N, Sharma A, Dewali S, Yadav M, Kumari R, Singh S, Mohapatra A, Pandey V, Rana N, Cunill JM (2022) Current status of pesticide effects on environment, human health and it’s eco-friendly management as bioremediation: a comprehensive review. Front Microbiol 17(13):962619. 10.3389/fmicb.2022.962619 10.3389/fmicb.2022.962619 PMC9428564 36060785

[CR112] Patra JK, Das G, Lee S, Kang SS, Shin HS (2018) Selected commercial plants: a review of extraction and isolation of bioactive compounds and their pharmacological market value. Trends Food Sci Technol 82:89–109

[CR59] Pereira L (2021) Macroalgae. Encyclopedia. 10.3390/encyclopedia1010017

[CR103] Pereira L, Cotas J (2023) Therapeutic potential of polyphenols and other micronutrients of marine origin. Mar Drugs 21(6):323 37367648 10.3390/md21060323 PMC10303569

[CR60] Pereira L, Cotas J (2024) Seaweed: a sustainable solution for greening drug manufacturing in the pursuit of sustainable healthcare. Explor Drug Sci 2:50–84. 10.3734/eds.2024.00036

[CR61] Perez-Vazquez A, Carpena M, Barciela P, Cassani L, Simal-Gandara J, Prieto MA (2023) Pressurized liquid extraction for the recovery of bioactive compounds from seaweeds for food industry application: a review. Antioxidants 12(3):612. 10.3390/antiox12030612 36978860 10.3390/antiox12030612 PMC10045370

[CR62] Poddar, Sourik. (2021). SEAWEED EXTRACT'S USE IN AGRICULTURE.10.5281/zenodo.5893885.

[CR91] Popp J, Pető K, Nagy J (2013) Pesticide productivity and food security. A review. Agron Sustain Dev 33:243–255

[CR63] Quitério E, Grosso C, Ferraz R, Delerue-Matos C, Soares C (2022) A critical comparison of the advanced extraction techniques applied to obtain health-promoting compounds from seaweeds. Mar Drugs 20(11):677. 10.3390/md20110677 36355000 10.3390/md20110677 PMC9695316

[CR64] Rafiquzzaman SM, Ahmed R, Lee JM, Noh G, Jo G, Kong I-S (2016) Improved methods for isolation of carrageenan from *Hypnea musciformis* and its antioxidant activity. J Appl Phycol 28:1265–1274

[CR89] Raghunandan BL, Vyas RV, Patel HK, Jhala YK (2019) Perspectives of seaweed as organic fertilizer in agriculture. In: Soil fertility management for sustainable development, pp 267–289

[CR65] Raja B, Vidya R (2023) Application of seaweed extracts to mitigate biotic and abiotic stresses in plants. Physiol Mol Biol Plants 29(5):641–661. 10.1007/s12298-023-01313-9 37363418 10.1007/s12298-023-01313-9 PMC10284787

[CR66] Rony ZI, Rasul MG, Jahirul MI, Mofijur M (2024) Harnessing marine biomass for sustainable fuel production through pyrolysis to support United Nations’ sustainable development goals. Fuel 358:130099

[CR67] Ruiz-Domínguez MC, Medina E, Salinas F, Bugueño W, Fuentes JL, Vílchez C, Garbayo I, Cerezal- MP (2022) Methodological optimization of supercritical fluid extraction of valuable bioactive compounds from the acidophilic microalga *Coccomyxa onubensis*. Antioxidants 11(7):1248. 10.3390/antiox11071248 35883739 10.3390/antiox11071248 PMC9312109

[CR108] Saberi Riseh R, Skorik YA, Thakur VK, Moradi Pour M, Tamanadar E, Noghabi SS (2021) Encapsulation of plant biocontrol bacteria with alginate as a main polymer material. Int J Mol Sci 22(20):11165 34681825 10.3390/ijms222011165 PMC8538305

[CR68] Samada LH, Tambunan USF (2020a) Biopesticides as promising alternatives to chemical pesticides: a review of their current and future status. Online J Biol Sci 20:66–76. 10.3844/ojbsci.2020.66.76

[CR69] Samada L, Tambunan U (2020b) Biopesticides as promising alternatives to chemical pesticides: a review of their current and future status. OnLine J Biol Sci 20:66–76. 10.3844/ojbsci.2020.66.76

[CR70] Satpati G, Sengupta S, Pal R (2022) Seaweeds: the ecological roles, the economic benefits and the threats for changing the carbon cycle. Springer, Cham

[CR71] Shukla PS, Borza T, Critchley AT, Prithiviraj B (2021) Seaweed-based compounds and products for sustainable protection against plant pathogens. Mar Drugs 19(2):59. 10.3390/md19020059 33504049 10.3390/md19020059 PMC7911005

[CR94] Sierra ARG, Amador-Castro LF, Ramírez-Partida AE, García-Cayuela T, Carrillo-Nieves D, Alper HS (2022) Valorization of Caribbean Sargassum biomass as a source of alginate and sugars for de novo biodiesel production. J Environ Manag 324:116364 10.1016/j.jenvman.2022.116364 36191503

[CR72] Smith HG, Roberts MJ, Pope WT (2018) Terpene based biopesticides as potential alternatives to synthetic insecticides for control of aphid pests on protected ornamentals. Crop Protect. 10.1016/j.cropro.2018.04.011

[CR73] Subbiah V, Xie C, Dunshea FR, Barrow CJ, Suleria HA (2022) The quest for phenolic compounds from seaweed: nutrition, biological activities and applications. Food Rev Int. 10.1080/87559129.2022.2094406

[CR74] Sultana P. Master’s Thesis. Chattogram Veterinary & Animal Sciences University; Chattogram, Bangladesh: 2019. Dietary Effects of Seaweed (*Hypnea musciformis*) on Growth Performance and Blood Parameters in Mice.

[CR75] Sundhar S, Shakila RJ, Jeyasekaran G, Aanand S, Shalini R, Arisekar U, Surya T, Malini NAH, Boda S (2020) Risk assessment of organochlorine pesticides in seaweeds along the Gulf of Mannar, Southeast India. Mar Pollut Bull 161(Pt B):111709. 10.1016/j.marpolbul.2020.111709 33038713 10.1016/j.marpolbul.2020.111709

[CR76] UNO (2024), United National conference on trade and development, An Ocean of Opportunities: The Potential of Seaweed to Advance Food, Environmental and Gender Dimensions of the SDGs, UNCTAD/DITC/TED/2024/1, ISBN: 978–92–1–003065–6 ,eISBN: 978–92–1–358834–5.

[CR77] Upadhyay H, Mirza A, Singh J (2020) Impact of biopesticides in sustainable agriculture. Springer, Singapore

[CR78] Uwineza PA, Waśkiewicz A (2020) Recent advances in supercritical fluid extraction of natural bioactive compounds from natural plant materials. Molecules 25(17):3847. 10.3390/molecules25173847 32847101 10.3390/molecules25173847 PMC7504334

[CR79] Wekre ME, Kåsin K, Underhaug J, Holmelid B, Jordheim M (2019) Quantification of polyphenols in seaweeds: a case study of *Ulva intestinalis*. Antioxidants 8(12):612. 10.3390/antiox8120612 31816918 10.3390/antiox8120612 PMC6943488

[CR80] Wells ML, Potin P, Craigie JS, Raven JA, Merchant SS, Helliwell KE, Smith AG, Camire ME, Brawley SH (2017) Algae as nutritional and functional food sources: revisiting our understanding. J Appl Phycol 29:949–982. 10.1007/s10811-016-0974-5 28458464 10.1007/s10811-016-0974-5 PMC5387034

[CR81] Wijesinghe WAJP, Jeon Y-J (2012) Enzyme-assistant extraction (EAE) of bioactive components: a useful approach for recovery of industrially important metabolites from seaweeds: a review. Fitoterapia 83:6–12. 10.1016/j.fitote.2011.10.016 22061659 10.1016/j.fitote.2011.10.016

[CR82] Wijesinghea JP, You JP (2012) Biological activities and potential industrial applications of fucose rich sulfated polysaccharides and fucoidans isolated from brown seaweeds: a review. Carbohydr Polym 88:13–20. 10.1016/j.carbpol.2011.12.029

[CR83] World Bank (2023a). Global Seaweed New and Emerging Markets. Report.

[CR84] Xu J, Liao W, Liu Y, Guo Y, Jiang S, Zhao C (2023) An overview on the nutritional and bioactive components of green seaweeds. Food Prod Process and Nutr 5(1):18. 10.1186/s43014-023-00132-5 10.1186/s43014-023-00132-5 PMC10026244 40476904

[CR113] Yang CF, Lai SS, Chen YH, Liu D, Liu B, Ai C et al (2019) Anti-diabetic effect of oligosaccharides from seaweed Sargassum confusum via JNK-IRS1/PI3K signalling pathways and regulation of gut microbiota. Food Chem Toxicol 131:110562 31181236 10.1016/j.fct.2019.110562

[CR115] Yang Y, Zhang M, Alalawy AI, Almutairi FM, Al-Duais MA, Wang J, Salama ES (2021) Identification and characterization of marine seaweeds for biocompounds production. Environ Technol Innov 24:101848

[CR85] Yao Y, Wang X, Chen B, Zhang M, Ma J (2020) Seaweed extract improved yields, leaf photosynthesis, ripening time, and net returns of tomato (*Solanum lycopersicum* Mill.). ACS Omega 5(8):4242–4249. 10.1021/acsomega.9b0415 32149254 10.1021/acsomega.9b04155 PMC7057707

[CR117] Zaharudin N, Tullin M, Pekmez CT, Sloth JJ, Rasmussen RR, Dragsted LO (2021) Effects of brown seaweeds on postprandial glucose, insulin and appetite in humans–A randomized, 3-way, blinded, cross-over meal study. Clin Nutr 40(3):830–838 32917417 10.1016/j.clnu.2020.08.027

[CR118] Zandi L, Makungu M, Munissi JJ, Duffy S, Puttreddy R, von der Heiden D et al (2020) Secoiridoids and Iridoids from Morinda asteroscepa. J Nat Prod 83(9):2641–2646 32852949 10.1021/acs.jnatprod.0c00447 PMC7522965

[CR86] Zhang QW, Lin LG, Ye WC (2018) Techniques for extraction and isolation of natural products: a comprehensive review. Chin Med 17(13):20. 10.1186/s13020-018-0177-x 10.1186/s13020-018-0177-x PMC5905184 29692864

[CR87] Zheng H, Zhao Y, Guo L (2022) A bioactive substance derived from brown seaweeds: phlorotannins. Mar Drugs 20(12):742. 10.3390/md20120742 36547889 10.3390/md20120742 PMC9785976

[CR120] Zhou QL, Wang Z, Chen WT, Liu XF, Cheong KL, Zou YX et al (2024) The structural characteristics, biological activities and mechanisms of bioactive brown seaweed polysaccharides: a review. J Funct Foods 119:106303

